# Multicenter Evaluation of Independent High-Throughput and RT-qPCR Technologies for the Development of Analytical Workflows for Circulating miRNA Analysis

**DOI:** 10.3390/cancers12051166

**Published:** 2020-05-05

**Authors:** Anna Babayan, Martin H. D. Neumann, Andrei Herdean, Jonathan M. Shaffer, Melanie Janning, Franca Kobus, Sonja Loges, Francesca Di Pasquale, Mikael Kubista, Martin Schlumpberger, Rita Lampignano, Thomas Krahn, Thomas Schlange, Markus Sprenger-Haussels, Klaus Pantel, Vera Kloten

**Affiliations:** 1 Department of Tumor Biology, University Medical Center Hamburg-Eppendorf (UKE), 20246 Hamburg, Germany; Anna.Babayan@qiagen.com (A.B.); m.janning@uke.de (M.J.); francakobus@yahoo.de (F.K.); Sonja.Loges@medma.uni-heidelberg.de (S.L.); pantel@uke.de (K.P.); 2 QIAGEN GmbH, 40724 Hilden, Germany; Martin.Neumann@qiagen.com (M.H.D.N.); Francesca.DiPasquale@qiagen.com (F.D.P.); Martin.Schlumpberger@qiagen.com (M.S.); Markus.Sprenger-Haussels@qiagen.com (M.S.-H.); 3 TATAA Biocenter AB, 411 03 Gothenburg, Sweden; Andrei.Herdean@uts.edu.au (A.H.); mikael.kubista@tataa.com (M.K.); 4 QIAGEN, Fredrick, MD 21703 USA; Jonathan.Shaffer@qiagen.com; 5 Department of Oncology, Hematology and Bone Marrow Transplantation with Section Pneumology, University Medical Center Hamburg-Eppendorf, 20246 Hamburg, Germany; 6 Division of Personalized Medical Oncology, German Cancer Research Center (DKFZ), 69120 Heidelberg, Germany; 7 Department of Personalized Oncology, University Hospital Mannheim, 68167 Mannheim, Germany; 8 Bayer AG, Pharmaceutical Division, Precision Medicine Markers, 42096 Wuppertal, Germany; rital@miltenyibiotec.de (R.L.); thomas.krahn@t-online.de (T.K.); thomas.schlange@bayer.com (T.S.)

**Keywords:** liquid biopsy, circulating miRNA, high-throughput, RT-qPCR, multicentric study

## Abstract

Background: Among emerging circulating biomarkers, miRNA has the potential to detect lung cancer and follow the course of the disease. However, miRNA analysis deserves further standardization before implementation into clinical trials or practice. Here, we performed international ring experiments to explore (pre)-analytical factors relevant to the outcome of miRNA blood tests in the context of the EU network CANCER-ID. Methods: Cell-free (cfmiRNA) and extracellular vesicle-derived miRNA (EVmiRNA) were extracted using the miRNeasy Serum/Plasma Advanced, and the ExoRNeasy Maxi kit, respectively, in a plasma cohort of 27 NSCLC patients and 20 healthy individuals. Extracted miRNA was investigated using small RNA sequencing and hybridization platforms. Validation of the identified miRNA candidates was performed using quantitative PCR. Results: We demonstrate the highest read counts in healthy individuals and NSCLC patients using QIAseq. Moreover, QIAseq showed 15.9% and 162.9% more cfmiRNA and EVmiRNA miRNA counts, respectively, in NSCLC patients compared to healthy control samples. However, a systematic comparison of selected miRNAs revealed little agreement between high-throughput platforms, thus some miRNAs are detected with one technology, but not with the other. Adding to this, 35% (9 of 26) of selected miRNAs in the cfmiRNA and 42% (11 of 26) in the EVmiRNA fraction were differentially expressed by at least one qPCR platform; about half of the miRNAs (54%) were concordant for both platforms. Conclusions: Changing of (pre)-analytical methods of miRNA analysis has a significant impact on blood test results and is therefore a major confounding factor. In addition, to confirm miRNA biomarker candidates screening studies should be followed by targeted validation using an independent platform or technology.

## 1. Introduction

Analysis of tumor-derived material in the context of liquid biopsy has gained substantial interest in the past decades as a powerful tool for minimally and non-invasive companion diagnostics [1,2].

Besides well-established analytes, like circulating tumor cells and circulating tumor DNA, circulating transcriptome, particularly non-coding RNA (ncRNA) emerged as potential source of valuable information for diagnosis, prognosis and prediction of treatment response in cancer patients [3,4]. The potential role of miRNAs as cancer biomarkers has attracted exponentially growing attention in the last ten years. miRNAs are about 22 nucleotides in length and regulate the expression of protein-coding genes by either degradation or specifically blocking of target mRNAs both, in the cell of origin, as well as in paracrine manner [5,6,7,8,9]. In blood and other body fluids cell-free miRNA (cfmiRNA) is present either in complexes with (lipo-) proteins or encapsulated in extracellular vesicles (EVs) [10,11].

At present, clinical application of liquid miRNA biomarkers remains uncertain. The reasons for the slow progress in the area is the lack of standardized pre-analytical and analytical workflows, as well as normalization strategies. Addressing the unmet need in the validation of complete workflows for material collection, miRNA and EV isolation, storage, analysis and respective normalization and interpretation of data, accompanied by rapid development of analytical technologies, is one of the aims of the IMI CANCER-ID consortium (https://www.cancer-id.eu/the-project/) for liquid biopsy-based technology evaluation. In our previous publication [12], we compared extraction efficiencies of five different protocols for cfmiRNA and two protocols for EV-related miRNA (EVmiRNA) isolation from plasma samples in a multicentric manner. Here, using the best performing methods, i.e., the miRNeasy Serum/Plasma Advanced and the ExoRNeasy Maxi kit, total miRNA and extracellular vesicle-derived miRNA samples from NSCLC patients (*n* = 27) and healthy volunteers (*n* = 20) were extracted and distributed to the participating sites for analysis. Data from the samples generated with the Toray three-dimensional (3D) Gene system, nCounter and QIAseq were centrally analyzed for an in-depth comparative data analysis (Screening study). Candidate miRNAs were selected for verification by two different quantitative PCR methods (miRCURY, two-tailed qPCR) in the same sample cohort of NSCLC patients and healthy controls (Validation study).

## 2. Results

### 2.1. Screening Study

#### 2.1.1. QIAseq Showed Higher Read Counts in Healthy Individuals and NSCLC Patients Compared to Hybridization Platforms

First, we compared the QIAseq miRNA library kit (sequencing platform) with the nCounter and Toray3 D (hybridization platforms) for the detection of cfmiRNA and EVmiRNA extracted from lung cancer patients and healthy individuals. The mapping distribution of different small RNAs measured with the QIAseq miRNA library kit is shown in Figure S1.

**Figure 1:**
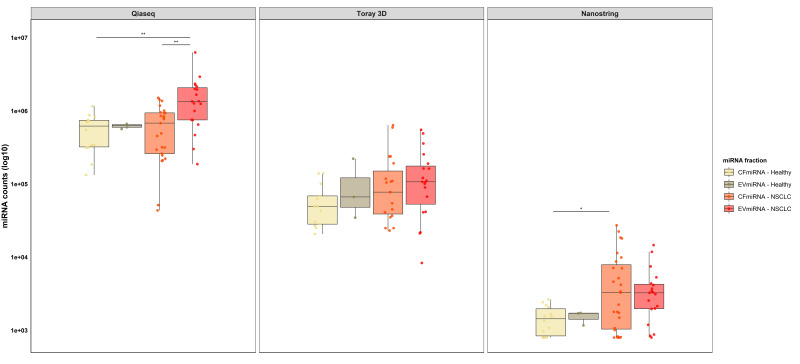
QIAseq reveals higher read counts in healthy individuals and NSCLC patients compared to hybridization platforms. Box plot analysis showing comparison of miRNA counts between QIAseq, Toray 3D and nCounter in cfmiRNA and EVmiRNA fractions. The horizontal line in each box represents the median; the whiskers indicate the range; statistical analysis was performed using one-way ANOVA where; ** *p* < 0.01, * *p* < 0.05.

Box plot analysis (Figure 1) shows the total read numbers in different miRNA fractions for each platform. QIAseq revealed the highest miRNA counts in all miRNA fractions compared to the hybridization platforms. Moreover, the QIAseq showed significantly (*p* < 0.01) higher read numbers in EVmiRNA of NSCLC patients compared to the cfmiRNA fraction of healthy controls and NSCLC patients. With the nCounter, we are able to show significantly (*p*< 0.05) higher read numbers in the cfmiRNA fraction, extracted from NSCLC patients, compared to healthy control samples. In more detail, sequencing of miRNA extracted from healthy individuals resulted in a mean of 5.66E+05 (± 3.01E + 05) and 6.20E + 05 (± 5.03E + 04) reads for cfmiRNA and EVmiRNA, respectively, while NSCLC patients showed a mean of 6.56E + 05 (± 4.41E + 05) for the cfmiRNA and 1.63E + 06 (± 1.36E + 06) for EVmiRNA fraction. Moreover, both Toray 3D and nCounter showed slightly higher mean miRNA counts in NSCLC patients (Toray 3D: 1.46E + 05 (± 1.78E + 05) and 1.58E + 05 (± 1.54E + 05) for the cfmiRNA and the EVmiRNA fraction, respectively; nCounter: 6.18E + 03 (± 7.32E + 03) and 4.05E + 03 (± 3.66E + 03) for the cfmiRNA and the EVmiRNA fraction, respectively) compared to healthy individuals (Toray 3D: 6.03E + 04 (± 4.13E + 04) and 1.08E + 05 (± 1.00E + 05) for the cfmiRNA and the EVmiRNA fraction, respectively; nCounter: 1.48E + 03 (± 6.49E + 02) and 1.54E + 03 (± 3.17E + 02) for the cfmiRNA, and the EVmiRNA fraction, respectively).

Of interest, dividing mean counts from NSCLC patients by mean counts from healthy control samples showed for the QIAseq 15.9% and 162.9% more cfmiRNA, and EVmiRNA miRNA counts, respectively, in NSCLC patients compared to healthy control samples. Among the hybridization platforms, Toray 3D showed 142.1% and 46.3% and the nCounter 317.6% and 163.0% more cfmiRNA and EVmiRNA miRNA counts, respectively, in NSCLC patients compared to healthy control samples.

#### 2.1.2. Systematic Comparisons Revealed Little Agreement between High-Throughput Platforms

To evaluate the differential expression concordance among individual platforms, we selected 26 miRNAs, including well-known control miRNAs. Surprisingly, results shown in Table 1 indicate that there is little agreement between the results obtained and that some miRNAs are detected with one technology, but none of the other. The hemolysis control miRNAs (miR-451a, miR-23a-3p) were detected by almost each platform without significantly differentiated expression between healthy controls and NSCLC patients. However, the QIAseq did not detect miR-451a in the EVmiRNA, as well as in the cfmiRNA fraction. Adding to this, miR-23a-3p was not detected by the Toray 3D platform in the cfmiRNA fraction. To our surprise, miR-30c-5p was detected with the Toray 3D in the EVmiRNA and with the QIAseq in the cfmiRNA fraction. Among all pre-selected miRNAs, the hybridization platforms nCounter and Toray 3D detected 35% and 50% of selected miRNAs in the EVmiRNA and 23%, and 31% in the cfmiRNA fractions, respectively. QIAseq detected only 15% of selected miRNAs in the EVmiRNA fraction, while 77% of selected miRNAs could be detected using cfmiRNA (Table 1).

In more detail, in the EVmiRNA fraction pre-selected miRNA biomarker candidates with significantly differentiated expression between healthy control and patients’ samples were found with the QIAseq (miR-190b and miR-1914-5p) and the nCounter (miR-144-3p, miR-191-5p, miR-199b-3p) platforms, showing no overlap. miR-5001-5p was shown to be detected by QIAseq and Toray 3D without significant expression between healthy control and patients’ samples. Surprisingly, commonly published and highly expressed miRNAs potentially relevant for diagnosis of NSCLC (for an overview see [13]), were not detected using QIAseq and Toray 3D.

In the cfmiRNA fraction, 42.3% candidate miRNAs (11 of 26 miRNAs) were detected by at least two platforms. In more detail, one miRNA (miR-122-5p) was detected with a significantly differentiated expression between sample groups by QIAseq and nCounter, while four miRNAs (miR-1914-5p, miR-144-3p, miR-671-5p, miR-4433a) were detected without a significantly differentiated expression between healthy control and patients’ samples. In addition, miR-16-5p and miR-186-5p were detected by QIAseq and nCounter showing a significant expression between sample groups (QIAseq) or not (nCounter). Furthermore, miR-1909-3p and miR-5739 were significantly expressed between healthy control and patients’ samples detected with QIAseq while no significantly, differentially expression was shown for Toray 3D. However, miR-5001-5p detected with Toray 3D revealed a significant differential expression between investigated cohorts, while no significance was shown for QIAseq (Table 1).

### 2.2. Validation Study

In the validation study, we compared the miRCURY with a two-tailed qPCR (qPCR platforms) for the detection of selected miRNAs (see Table 1). In brief, miR-23a-3p and miR-451 (hemolysis controls) were detected with both methods, while miR-30c-5p (negative control) was detected by the miRCURY only. In addition, miR-124-3p (negative control) was not detectable with both qPCR platforms. Among all miRNAs, miRCURY detected 62% and 77% of pre-selected miRNA candidates, while the two-tailed qPCR detected 42% and 65% in the EVmiRNA, and cfmiRNA fraction, respectively. Adding to this, 35% (9 of 26) of selected miRNAs in the cfmiRNA and 42% (11 of 26) in the EVmiRNA fraction were differentially expressed by only one qPCR platform, while about half of the miRNAs 54% (14 of 26) were concordant for both platforms.

**Figure 2:**
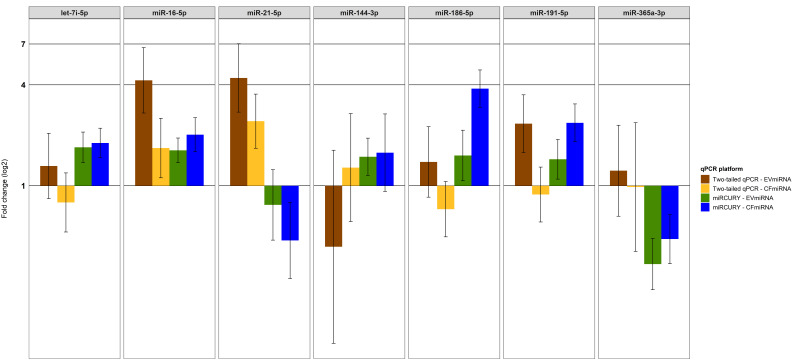
Two-tailed qPCR and miRCURY lead to different miRNA profiles. The fold change (2^-∆∆Cq^) between lung cancer patients and control samples was calculated using global normalized ∆Cq- values and compared between qPCR platforms. Error bars show the limits of the 95%CI.

Next, we performed a global normalization by subtracting the mean Cq-value of all miRNAs in a given sample per each platform and miRNA fraction. In addition, we calculated and compared the fold change (2-∆∆Cq) between lung cancer patients and control samples (Figure 2). About 32% (7 of 22) of selected miRNAs were detected by both platforms showing a different regulation trend for some candidate miRNAs. While only miR-16-5p was upregulated in cfmiRNA and EVmiRNA shown by miRCURY and two-tailed qPCR, especially two-tailed qPCR showed a divergent regulation between the cfmiRNA and EVmiRNA fractions (either down-or upregulation). Of interest, miR-21 was shown to be upregulated in the two-tailed qPCR and downregulated in the miRCURY platform.

## 3. Discussion

In this study, we utilized the best performing protocols for the extraction of cfmiRNA and EVmiRNA as already shown by Kloten et al. [12] within the CANCER-ID consortium. Here, five miRNA detection platforms were compared on isolated miRNA fractions from NSCLC patients and healthy control samples revealing low concordance between quantification technologies.

In recent years, several studies [12,14,15,16] underlined the unmet need of standardized strategies in the (pre)-analytical phase to minimize the effects of variables, including blood collection, sample handling, as well as miRNA isolation and analysis. The four platforms evaluated in this study included a small RNA-sequencing protocol, Toray 3D, nCounter and two different qPCR approaches (miRCURY, two-tailed qPCR). As yet, systematic comparisons between these quantification platforms have demonstrated their utility for research studies [15,16]. However, clinical application of liquid miRNA biomarkers remains uncertain due to analytical limitations of each method. To our knowledge, a multicentric study using cell-free and extracellular-derived miRNA fractions from lung cancer patients and healthy individuals has not been previously performed. These plasma miRNA fractions allowed us to compare how the platforms performed on biological samples.

In the present study, QIAseq exhibited higher miRNA counts in all sample groups, compared to the two tested hybridization platforms, showing significantly more miRNA counts in the EVmiRNA fraction of NSCLC patients, compared to cfmiRNA from NSCLC patients and healthy control samples. Furthermore, in our hands, the nCounter yielded a much smaller fraction of miRNAs detected above background compared to the other two platforms, which could be due to lower sensitivity of this platform, which is consistent with reports of previous studies [15,16]. However, the detection rate is a combination of sensitivity, detection cut-off and number of assays or probes available on the platform. Furthermore, it is important to recognize that all methods use different input amounts which may limit the direct comparison between technologies. Taking these technical features into account, our results within the screening phase of this study are consistent with the results of previous studies [15,16]. Adding to this, we could show higher miRNA counts in the cfmiRNA and EVmiRNA fractions from NSCLC patients compared to healthy control samples for all three platforms which is beyond the scope of other studies. Of interest, platforms with overall lower read numbers, like the nCounter, showed proportionally higher read numbers in lung cancer patients compared to healthy control samples. We selected a set of 28 miRNAs relevant in NSCLC [13] and identified them as differentially expressed by QIAseq and Toray 3D. However, the concordance of results obtained by these two technologies was low. Similarly, Mestdagh et al. [16] showed that only 3% of the selected miRNAs (2 of 66) were shown to be differentially expressed by all investigated platforms (*n* = 12), including the nCounter used in this study, while almost half of the miRNAs (48%) were concordant at least for half of the platforms.

In the validation phase, 35% (9 of 26) of selected miRNAs in the cfmiRNA and 42% (11 of 26) in the EVmiRNA fraction were differentially expressed by at least one qPCR platform; about half of the miRNAs (54%) were concordant for both platforms in the cfmiRNA fraction only. Adding to this, both qPCR platforms detected more miRNAs (miRCURY: 77% (20 of 26); 2-tailed qPCR: 65% (17 of 26)) candidates in the cfmiRNA compared to the EVmiRNA fraction. In our hands, miRNA candidates potentially relevant in NSCLC diagnosis and prognosis (i.e., miR-16-5p, miR-20a-5p, miR-21-5p, miR-125b-5p) were mainly detected in the cfmiRNA fraction using QIAseq and the miRCURY platform. Contrary, those miRNAs could only be detected in the EVmiRNA fractions with the miRCURY, but not with the QIAseq, which might be due a lower yield of the mentioned miRNAs in extracellular vesicles. Interestingly, miRNA candidates particularly detected with both the QIAseq and Toray 3D in either the cfmiRNA or the EVmiRNA fraction were barely detected by any of the two qPCR platforms. In this context, Godoy and colleagues recently showed that small RNA-seq was prone to detect reads that mapped to miRNAs which were not present in a synthetic equimolar pool of miRNAs. The authors assumed errors introduced during PCR or sequencing or caused by contamination for this ‘‘not present’’ miRNAs [15]. This strongly demonstrates that screening studies should be followed by targeted validation using an alternative platform or technology.

Our results confirm previous studies observing higher miRNA read counts using small RNA-seq and a concordance lower than expected across technology platforms [15,16]. One limitation of our study was the investigation of a heterogenous NSCLC patients’ cohort, including patients at different stages of their disease before and during systemic or radiotherapy as well as before and after surgery. miRNA evaluation in clinical subgroups would be quite interesting, but this was beyond the scope of this technical validation study. Finally, our control cohort was not age-matched to NSCLC patients, and is the reason why caution should be exercised in interpreting the fold changes between NSCLC patients and healthy control samples for different miRNA fractions and qPCR platforms.

## 4. Materials and Methods

### 4.1. Study Design

For this study, whole blood from 27 non-small cell lung cancer patients (NSCLC) was collected at the University Medical Center Hamburg-Eppendorf. Blood was collected before, and during, the treatment of patients. All patients gave written informed consent for retention and analysis of their blood for research purposes (local ethical review board of the Ärztekammer Hamburg, approval PV5392). An overview of patients’ characteristics is given in Table 2. Similarly, 20 healthy donors (age 40–65 years) were enrolled after giving written informed consent at Clinical Research Services GmbH (CRS) Wuppertal (local ethical review board of the medical association Nordrhein, ref no. A 18/009). Based on the results of the previous miRNA ring trial [10], the two best performing kits, miRNeasy Serum/Plasma Advanced and ExoRNeasy (both QIAGEN), were used for isolation of cell-free, and EV-associated miRNA from plasma, respectively. Next, systematic comparison of different NGS-based small RNA sequencing and microarray platforms was designed as a multicentric ring study (herein after referred to as screening study). Validation of the identified miRNA candidates was performed using a two-tailed RT-qPCR [17] and a customized miRCURY LNA miRNA assay (QIAGEN) (herein after referred to as validation study).

### 4.2. Plasma Preparation

Blood samples from all blood donors were obtained by venipuncture. Blood was collected in 7.5 mL K3EDTA S-Monovette (Sarstedt, Germany). Per patient 1–4 tubes were collected. Blood tubes remained at ambient temperature until plasma was generated within 2 h after blood draw. Whole blood was centrifuged at 2500× *g* for 10 min. Plasma was carefully transferred into new 15 mL conical tubes (Greiner Bio-One, Kremsmünster, Austria) and centrifuged a second time at 2500× *g* for 10 min to remove cellular debris. 2.75–12.3 mL supernatant was collected and immediately frozen at −80 °C.

### 4.3. Isolation of cfmiRNA and Extracellular Vesicle Derived miRNA (EVmiRNA)

Prior to extraction, plasma samples were thawed in a waterbath at 37 °C until thawed and vortexed for 1 min. Isolation of total cfmiRNA from plasma from NSCLC patients and healthy individuals was done with the miRNeasy Serum/Plasma Advanced kit (Cat No./ID: 217204, QIAGEN, Germany) according to the manufacturer’s protocol. For each individual plasma sample, 6 preparations were done with 300 µL input and 17 µL elution volume each. Eluates were pooled for each donor.

In case the remaining volume of plasma was ≥ 2500 µL, additional isolation of exosomal RNA was performed (19 out of 27 NSCLC patients’ samples) using the ExoRNeasy Maxi kit (Cat No./ID: 77064, QIAGEN, Germany) according to the manufacturer’s instructions. Total RNA, including EVmiRNA, was eluted in 73 µL of RNase-free water.

Finally, eluates of cfmiRNA and EV-associated miRNA were aliquoted in 96-well plates in accordance with requirements of the respective analytic technology and immediately frozen at −80 °C until being shipped on dry ice to the participating research sites performing the miRNA analyses.

### 4.4. Small RNA Sequencing Platforms

#### QIAseq miRNA Library Kit

NGS libraries were prepared from 5 µL aliquots using the QIAseq miRNA Library kit (QIAGEN). Libraries and NGS procedure were performed as previously described [12].

### 4.5. Hybridization Platforms

#### 4.5.1. NanoString nCounter Assay

Analysis of the eluates of all samples was performed using the nCounter Analysis System (Nanostring Technologies) and the nCounter Human v3 miRNA Panel (Catalog #CSO-MIR3-12). The panel comprises 798 unique miRNA barcodes and probes for several reference genes (mRNA), e.g., Ribosomal protein L10 (RPL10), beta-actin (ACTB), beta-2-microglobulin (B2M), glyceraldehyde 3-phosphate dehydrogenase (GAPDH), and ribosomal protein L19 (RPL19). Additionally, control miRNAs from Arabidopsis thaliana (ath-miR159a), Caenorhabditis elegans (cel-miR-248 and -miR254), and Oryza sativa (osa-miR 414 and -442) as well as positive and negative controls are incorporated in the nCounter code sets. RNA was prepared and run according to the manufacturer’s protocol (nCounter® miRNA Expression Assay) in a total of 6 runs within 4 days. In total, 3 µL of eluate was loaded. All samples passed the system’s quality controls. Hybridization time was 16–18 h, and all counts were detected by scanning in HIGH mode. The raw miRNA data were analyzed using the nCounter nSolver analysis software version 3.0. Background correction was performed by subtracting the mean + 2x standard deviations of negative control as a cut-off.

#### 4.5.2. Toray 3D

Microarray analysis of the RNA samples was performed using the 3D-Gene miRNA microarray platform (Toray Industries, Tokio, Japan) with the Human miRNA Microarray ver.21 chip (Toray Industries, Tokio, Japan). The analysis was performed according to manufacturer’s instructions.

### 4.6. Quantitative PCR Platforms

#### 4.6.1. miRCURY

The method was used as described in the manufacturer´s handbook for Exosomes, Serum/Plasma and Other Biofluid Samples. Briefly, the combined poly-adenylation and the reverse transcription was performed using the miRCURY LNA RT Kit (QIAGEN). A total of 1 µL of the eluates was used in a 20 µL cDNA reaction. A BioRad T1000 touch thermocycler was used for this step with the following temperature program: 60 min at 42 °C, 5 min at 95 °C and 4 °C forever.

Quantitative PCR (qPCR) was performed using the miRCURY LNA miRNA PCR Assays in combination with the miRCURY LNA SYBR® Green PCR Kit (QIAGEN). The whole cDNA reaction was diluted 1:30 in RNAse-free water. 3 µL of the diluted cDNA was used in a 10 µL qPCR. qPCR was performed in duplicates in a 384-well plate using a BioRad CFX 384 qPCR instrument with the following temperature cycling: 95 °C for 2 min, 40 cycles of 95 °C for 10 s, 56 °C for 60 s, followed by melting-curve analysis. A list of analyzed miRNAs can be found in Table S1.

#### 4.6.2. Two-Tailed qPCR

The experimental procedure is based on previous publications [17,18] with minor modifications. Reverse transcription (RT) reactions were performed with the TATAA GrandScript cDNA FreePrime Kit in a total reaction volume of 10 μL. A BioRad T100 thermocycler was used for this step with the following temperature program: 45 min at 42 °C, 5 min at 85 °C and then held at 4 °C. cDNA was further diluted by adding 40 µL of nuclease-free water. Quantitative PCR (qPCR) was performed with TATAA SYBR® GenMaster Mix in a total volume of 10 μL, out of which 2 µL were the diluted cDNA. qPCR was performed in duplicates in a 384-well plate using a BioRad CFX 384 qPCR instrument with the following temperature program: 95 °C for 30 s, 40 cycles of 95 °C for 5 s, 60 °C for 15 s, and 72 °C for 10 s, followed by melting-curve analysis. All pipetting steps were performed using an Eppendorf epMotion 5070 pipetting robot. Concentration of each primer in final reactions were 400 nM for qPCR primers and 50 nM for RT primers.

### 4.7. Data Normalization

Sequencing (QIAseq) as well as hybridization (NCounter, Toray 3D) raw data were pre-processed according to each platform and further analyzed using the MultiD GenEx software 7.0.1.473. Data normalization consisted of the following steps: (i) miRNA without any counts in all samples were removed, (ii) all remaining values were converted to log2, (iii) missing values were replaced with -1, and (iv) data was global normalized (cfmiRNA) or normalized using Normfinder (EVmiRNA).

Raw Cq-values of each qPCR platform (miRCURY, Two-tailed qPCR) were normalized by subtracting the mean Cq-value of all miRNAs in a given sample. Next, we calculated the fold change (2-∆∆Cq) between lung cancer patients and control samples by using the mean normalized Cq-value of all samples for a given miRNA as a calibrator.

Raw data of all investigated platforms can be found in Tables S2 (Screening study) and S3 (Validation study).

### 4.8. miRNA Candidate Selection

We selected several miRNAs for comparison of different high-throughput technologies and for further evaluation in the validation study. miRNA candidates were included according to the following criteria:

(i) Control miRNAs (i.e. hemolysis controls miR-451a and miR-23a-3p, as well as negative controls widely found in urine samples (miR-30c-5p) and cerebrospinal fluid (CSF) (miR-124-3p)).
(ii) miRNAs relevant for NSCLC diagnosis (i.e. miR-16-5p, miR-20a-5p, miR-21-5p, miR-125b-5p).
(iii) Highly expressed miRNAs detected with QIAseq and Toray 3D.
(iv) miRNAs which were statistically significant, differentially expressed in healthy control vs. patient samples (at least with one method).

Each platform also included platform-specific controls.

### 4.9. Statistical Analysis

The R (version 3.6.1, The R Foundation for Statistical Computing, Vienna, Austria) and R Studio software (R Studio, Boston, MA, USA) were used for statistical analysis and data visualization. Statistical comparisons were performed using student’s *t*-test and two-way ANOVA. Two-sided tests with *p* < 0.05 were considered statistically significant. Visualization of results in box plots and fold change was carried out using the package ggplot2. The 95%CI were calculated using the ∆∆Cq-values.

## 5. Conclusions

In conclusion, this study provides a comparison of cell-free and extracellular vesicle-derived miRNAs, relevant in NSCLC, using widely used detection platforms. As highlighted in this study, we found differential miRNA expression between cancer patients and controls. However, the overlap between the results obtained by the different technologies was small, thereby demonstrating that miRNA results, obtained with different technologies in different studies, cannot be directly compared. This explains the heterogeneity and lack of reproducibility of published results, which highlights the urgent need for standardized technologies. It also calls for caution when using miRNA data for identification or interpretation of pathways deregulated in cancer patients, which should not be done without orthogonal assay validation.

## Figures and Tables

**Table 1:** Summary of different miRNA expression in healthy vs. NSCLC in the screening and validation phase for selected miRNAs.

miRNA	EVmiRNA					cfmiRNA					
	QIAseq	nCounter	Toray 3D	miRCURY	2-Tailed qPCR	QIAseq	nCounter	Toray 3D	miRCURY	2-Tailed qPCR	
	*Screening Study*			*Validation Study*		*Screening Study*			*Validation Study*		miRNA Class
**miR-23a-3p**	0.076	0.059	0.398	0.000 *n*	0.012 *n*	0.120	0.306	-	0.317	0.001 *n*	Hemolysis controls
**miR-451a**	-	0.363	0.297	0.002 *n*	0.005 *n*	-	0.072	0.235	0.001 *n*	0.001 *n*	
**miR-30c-5p**	-	-	0.004 H	0.013 *n*	n*	0.028 *n*	-	-	0.174	-	Negative controls
**miR-124-3p**	-	-	-	-	n*	-	-	-	0.419	0.348	
**miR-16-5p**	-	0.386	-	0.000 *n*	0.001 *n*	0.000 *n*	0.498	-	0.004 *n*	0.004 *n*	Potentially relevant for NSCLC diagnosis
**miR-20a-5p**	-	-	-	0.000 *n*	-	0.220	-	-	0.135	-	
**miR-21-5p**	-	0.073	-	0.199	0.007 *n*	0.000 *n*	-	-	0.203	0.001 *n*	
**miR-125b-5p**	-	-	-	0.004 *n*	n*	0.138	-	-	0.184	0.067	
**let-7i-5p**	-	-	-	0.303	0.159	0.000 *n*	-	-	0.166	0.003 *n*	Commonly found in "QIAseq cfmiRNA" and "miRCURY cfmiRNA"
**miR-122-5p**	-	-	-	0.023 *n*	-	0.005 *n*	0.024 *n*	-	0.185	-	
**miR-186-5p**	-	0.210	-	0.013 *n*	0.005 H	0.043 H	0.477	-	0.033 *n*	0.002 *n*	
**miR-190b**	0.022 *n*	-	-	-	-	0.000 *n*	-	-	0.150	-	
**miR-191-5p**	-	0.030 *n*	-	0.000 *n*	0.004 *n*	0.120	-	-	0.199	-	
**miR-199b-3p**	-	0.014 *n*	-	0.021 *n*	-	0.000 *n*	-	-	0.227	-	
**miR-211-3p**	-	-	0.211	n*	-	-	-	-	n*	-	
**miR-365a-3p**	-	0.444	0.166	0.264	0.342	-	-	-	0.176	0.468	
**miR-144-3p**	-	0.047 H	-	0.184	-	0.278	0.061	-	0.013 *n*	0.131	Commonly found in "QIAseq cfmiRNA" and "Toray cfmiRNA"
**miR-671-5p**	-	-	0.360	0.001 *n*	n*	0.194	-	0.345	0.077	0.444	
**miR-1909-3p**	-	-	0.446	-	0.131	0.000 H	-	0.224	0.020 *n*	0.292	
**miR-1914-5p**	0.006 *n*	-	-	-	0.442	0.484	-	-	0.154	0.029 H	
**miR-3195**	-	-	0.500	0.019 *n*	n*	-	-	0.336	0.098	0.366	
**miR-5739**	-	-	0.403	-	-	0.012 H	-	0.257	-	n*	
**miR-3185**	-	-	0.058	n*	0.115	0.031 H	-	-	-	0.488	
**miR-4433a**	-	-	0.289	n*	n*	0.352	-	0.353	n*	0.423	
**miR-5001-5p**	0.204	-	0.281	-	n*	0.130	-	0.050 *n*	-	0.311	
**miR-6795-5p**	-	-	0.067	-	-	-	-	0.136	-	-	
**miRNAs detected, %**	15	35	50	62	42	81	23	31	77	65	

Numbers indicate *p* values of statistical analysis (Student’s *t*-test) between healthy control and patient samples; green colored boxes indicate statistical significant expression in healthy control vs. patient samples; blue colored boxes indicate detected miRNAs which are not statistically significantly, differentially expressed in healthy control vs. patient samples; “-“ indicates that the miRNA was not detected; “*n*”—upregulated in NSCLC; “H”—upregulated in healthy; “n*”—detected only in NSCLC. Data was analyzed using GenEx 7; QIAseq: cfmiRNA and EVmiRNA data were normalized using Normfinder, miRNAs without counts and with less than 35 counts were removed. nCounter: cfmiRNA was global normalized; EVmiRNA data was normalized using Normfinder, miRNAs without counts were removed. Toray 3D: cfmiRNA and EVmiRNA data were normalized using Normfinder, miRNAs without counts were removed. miRCURY: data was normalized to UniSp6 spike-in; 2-tailed qPCR: data was not normalized.

**Table 2:** Patients’ characteristics.

Patients Characteristics	n	%
	27	
**Median age (range)**	65 (52–82)	
**Sex**		
Male	17	63.0
Female	10	37.0
**Histology**		
Adeno	25	92.6
Squamos	2	7.4
Missing information	0	0
**Tumor Size**		
T 1–2	8	29.6
T 3–4	15	55.6
Missing information	4	14.8
**Lympnode Metastasis**		
*n* neg	6	22.2
*n* pos	16	59.3
Missing information	5	18.5
**Distant Metastasis**		
M neg	7	25.9
M pos	18	66.7
Missing information	2	7.4
**Smoking status**		
smoker	10	37.0
never smoker	5	18.5
former smoker	3	11.1
Missing information	9	33.3
**Mutations**		
EGFR	6	22.2
KRAS	7	25.9
TP53	6	22.2
Missing information or other mutations	8	29.6
**Therapy (at blood draw)**		
Chemotherapy	9	33.3
Chemoradiotherapy	3	11.1
check point inhibitor	10	37.0
tyrosine kinase inhibitor	5	18.5
**Therapy Response (at the next follow up)**		
Stable disease	1	3.7
Partial response	0	0
Complete response	1	3.7
Progression disease	15	55.6
Missing information	10	37.0

## Supplementary Materials

The following are available online at https://www.mdpi.com/2072-6694/12/5/1166/s1, Figure S1: Mapping distribution of different small RNAs measured with the QIAseq miRNA library kit. Table S1: Information on miRNAs analyzed with the custom miRCURY LNA miRNA PCR Assays. Table S2: Raw data of investigated sequencing and hybridization platforms. Table S3: Raw data of investigated qPCR platforms.
